# Healthcare Technology Management (HTM) of mechanical ventilators by clinical engineers

**DOI:** 10.1186/2052-0492-2-27

**Published:** 2014-04-15

**Authors:** Jun Yoshioka, Masaki Nakane, Kaneyuki Kawamae

**Affiliations:** Department of Clinical Engineering, Yamagata University Hospital, 2-2-2 Iidanishi, Yamagata, 990-9585 Japan; Department of Anesthesiology, Yamagata University Faculty of Medicine, 2-2-2 Iidanishi, Yamagata, 990-9585 Japan

**Keywords:** Clinical engineers, Mechanical ventilator, Safe management

## Abstract

Mechanical ventilator failures expose patients to unacceptable risks, and maintaining mechanical ventilator safety is an important issue. We examined the usefulness of maintaining mechanical ventilators by clinical engineers (CEs) using a specialized calibrator. These evaluations and the ability to make in-house repairs proved useful for obviating the need to rent ventilators which, in turn, might prove faulty themselves. The CEs' involvement in maintaining mechanical ventilators is desirable, ensures prompt service, and, most importantly, enhances safe management of mechanical ventilators.

## Letter to the Editor

It is important to ensure appropriate quality control of mechanical ventilatory support. Medical devices in the hospital such as mechanical ventilators and anesthesia machines have been managed by doctors and nurses throughout the world [1–3]. In Japan, doctors and nurses have performed the maintenance of mechanical ventilators in the past [4]. Faulty mechanical ventilators were repaired by the distributor. However, since the late 1990s, clinical engineers (CEs) who have a certificate of completion and have completed established course requirements now perform the maintenance of mechanical ventilators in hospitals. The aim of this letter was to investigate the effectiveness of maintaining mechanical ventilators by CEs using a specialized calibrator in hospitals.

In our hospital, 30 mechanical ventilators (5 models) were used. These were all evaluated using a specialized PTS-2000 calibrator (Puritan-Bennett, Mansfield, MA, USA) at the time of after-use inspections and periodic testing, calibration, or maintenance. For each ventilator, we evaluated the following parameters: inspiratory flow, tidal volume, oxygen concentration, peak inspiratory pressure (PIP), positive end-expiratory pressure (PEEP), respiratory rate, and inspiratory time. Mechanical ventilators that were out of the acceptable ranges were classified as faulty. Faulty mechanical ventilators were repaired by calibration and replacing sensors and/or circuit boards.

We systematically checked mechanical ventilators about 2,500 times using a PTS-2000 calibrator during the period from January 2004 to December 2010, and there were a total of 151 cases of suspected mechanical ventilator failure. The number of faulty ventilators was 90 for ventilatory volume, 39 for oxygen concentration, and 22 for malfunctions. The faulty ventilators needing valve and/or sensor replacement were repaired in-house by CEs. The rare, seriously malfunctioning ventilators (there were a total of 12 cases of circuit board failure) were sent to the distributor for repair. Regarding response times for valve and/or sensor replacement, in-house repairs were completed immediately. However, if a machine needed to be repaired to the distributor, it takes from 2 h to half a day, as there are no distribution centers in Yamagata prefecture.

The number of mechanical ventilators rented has increased each year (198, 246, 288, 268, 313, 391, and 573 from 2004 to 2010). However, minor problems during daily practice that can be handled on the spot have been reduced because of after-use maintenance of mechanical ventilators by CEs (62, 60, 49, 47, 25, 10, and 12 from 2004 to 2010). Major failures (which necessitated a change-out of the mechanical ventilator) have been reduced because of after-use maintenance of mechanical ventilators by CEs (17, 11, 5, 3, 0, 4, and 0 from 2004 to 2010). Until 2003, CEs only checked and fixed broken ventilators. Most of the parts that were exchanged due to ventilator failure were batteries and sensors, parts that are recommended for periodic replacement. In the present study, consumable parts like batteries and sensors, which deteriorate over time, were at fault in the majority of cases. Therefore, we have routinely checked consumable parts, such as batteries and sensors, by using the PTS-2000 calibrator during inspections. And we have actively conducted calibrations and replaced consumable parts. Preventive inspection by CEs has decreased faulty ventilators. Thus, the involvement of CEs has improved mechanical ventilator safety.

During the 1980s, general-purpose-type mechanical ventilators were in use. Their operating principle was simple, and mechanical ventilators at that time had few circuit boards and/or sensors [5]. These mechanical ventilators were rugged and did not break down easily. Operator error accounted for most of the sudden malfunctions of mechanical ventilators [6, 7]. However, over the last two decades, technological developments have led to significant improvements in the performance of mechanical ventilators [8–11]. Modern critical care mechanical ventilators have circuit structures with numerous CPU boards and sensors [12]. Therefore, these mechanical ventilators have more failure points that increase the likelihood of failure. Blanch reported that spending an immense amount of time and cost could secure the reliability of an apparatus, but it was hard to anticipate the deterioration of mechanical ventilators; their safety and reliability will change significantly as ventilators are used or age [13]. The use of the PTS-2000 calibration analyzer has contributed to the discovery of previously undetected malfunctions in the new generation of high-performance mechanical ventilators. In this way, evaluating ventilation and carrying out in-house repairs has proved to be effective for obviating the chance of renting faulty units. Clinical engineering has decreased medical device failure which exposes patients to potentially harmful risks.

The most important item should be the checking of mechanical ventilators by CEs. CEs are certainly specialists in medical devices, and their involvement in maintaining mechanical ventilators is logical for the hospital, prompt, and most importantly safe. CEs fill an essential role for the safe operation of mechanical ventilators, and, more importantly, the CEs technology provides safe maintenance for many hospitals.
